# The Implementation and Optimization of Neonatal Epidural Analgesia in a Tertiary Care Hospital: A Technical Report

**DOI:** 10.7759/cureus.60657

**Published:** 2024-05-20

**Authors:** Timothy Hudson, Svetoslav M Saev, Mary Saev, Claudia Nadernejad

**Affiliations:** 1 Pediatric Anesthesiology, Helen DeVos Children’s Hospital, Grand Rapids, USA; 2 Internal Medicine, Trinity Health Muskegon, Grand Rapids, USA; 3 Pediatric Surgery, Corewell Health, Grand Rapids, USA; 4 School of Science, Indiana University–Purdue University Indianapolis, Indianapolis, USA; 5 Public Health, Walden University, Minneapolis, USA; 6 Obstetrics and Gynecology, Saint James School of Medicine, The Quarter, AIA; 7 Neonatology, Helen DeVos Children’s Hospital, Grand Rapids, USA

**Keywords:** ultrasound, caudal, ropivacaine, analgesia, epidural, neonatal

## Abstract

Effective analgesic therapy in neonates continues to be fundamental for improving quality of life and decreasing the need for further medical intervention. When pain is not well controlled in the neonatal intensive care setting, we see an increased use of sedation pharmaceuticals, mechanical ventilation, and altered somatosensory development, among other complications. Currently, there is no standardized protocol addressing effective pain management while decreasing the need for further sedation. In this article, we seek to demonstrate how our institution standardized and implemented the utilization of epidural analgesia in neonates as the preferred method of pain management for open thoracic and abdominal surgeries.

## Introduction

Postoperative analgesia in the neonatal population is complicated by high sensitivity to opioids and sedative medications, resulting in respiratory depression. As such, major operations requiring significant dosing of these medications for postoperative analgesia frequently necessitate maintenance of mechanical ventilation to ensure cardiopulmonary stability. Epidural analgesia using dilute, plain local anesthetic solutions, on the other hand, affords the opportunity to significantly decrease the need for depressive, systemic medications and, thus, reliance on ongoing mechanical ventilation. In addition to reducing ventilator dependence, numerous studies demonstrate improved pain scores despite significantly lowered opioid requirements with epidural analgesia [1,2]. Furthermore, tolerance to opioids and protracted neurological hypersensitivity are associated with inadequate analgesia in the neonatal period [3-5].

The primary barrier to placing epidural catheters in neonates is the technical difficulty of safely placing them. The traditional approach of using loss of resistance to needle insertion at the desired vertebral level is a challenge due to the still-developing spinal anatomy of the neonate and entry site leakage due to shallow depths to the epidural space. Lastly and most importantly, there is the concern of causing spinal cord injury due to the aforementioned factors.

The caudal approach (i.e., sacral hiatus entry) to the thoracic epidural space was initially described as a viable alternative to the direct placement level in 1988 [6]. Accessing the epidural space remotely to the body of the spinal cord obviates the concern of needle damage to the body of the spinal cord while allowing access to the epidural space. Epidural supplies explicitly designed for small babies have only recently become available. It is reasonable to understand why a quaternary children’s hospital, as recently as 2013, had little recent experience with neonatal epidurals [7]. In 2014, medical supply manufacturer Epimed™ began mass production of the first spring-wound, styletted epidural catheter and needles designed explicitly for small babies. Before 2014, adult supplies were required for epidural analgesia in neonates.

In light of the novelty of neonatal epidural analgesia for most of our pediatric anesthesiologists and neonatalogists, we initiated a protocol that standardizes the placement and management of epidural catheters that all pediatric anesthesiologists in our practice would be comfortable with. Additionally, we aimed to mitigate site leakage, confusion regarding pharmacologic management, and inaccurate vertebral localization. These issues had been recurrent barriers to uncomplicated neonatal epidural analgesia.

This implementation was a multidisciplinary collaboration between pediatric anesthesiology, pediatric surgery, and neonatology. What follows is a pragmatic description of how we have successfully implemented the use of reliable epidural catheters in neonates, intending to help other centers looking to improve neonatal analgesia.

## Technical report

Before the procedure, informed permission for anesthesia and epidural placement was discussed with the patient’s parents or medical decision-makers. The procedure overview, risks, benefits, associated complications, and alternative treatments were addressed. Following the informed decision of the responsible parties, consent was obtained for the procedure and filed in the patient’s medical records. The Institutional Review Board of Corewell Health, Grand Rapids, Michigan, reviewed the project and protocol on March 6, 2023, and it was determined that it is exempt from research per 45 CFR Part 46.104(d) category 4. The waiver of HIPAA authorization was approved per Article 45 CFR 164.512(i)(2)(ii).

Placement

**Figure 1:**
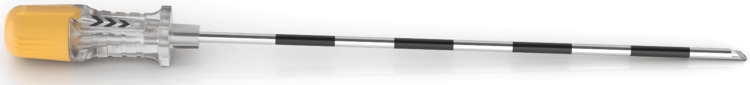
Example of a 20-gauge Crawford epidural needle. A Crawford needle is used for the insertion of a spinal catheter into the subarachnoid space for procedures requiring spinal anesthesia or drug administration. Some needles feature a curved design and a sharp beveled tip, while others have a rounded rear heel and short bevel to resist shearing from the catheter tip during insertion. These catheters facilitate insertion through the skin, subcutaneous tissue, and spinal ligaments into the desired spinal level. It typically has markings to help gauge depth and is available in various sizes to accommodate different patient anatomies and procedural requirements.

The caudal space was selected as the default insertion site for neonates because of the extensive experience our anesthesiologists have with single-shot caudal blocks and to avoid the risk of spinal cord injury with epidural needle insertion. Positioning the patient in as much fetal position (hip, lumbosacral, and cervical flexion) as possible is known to pull the terminus of the thecal sac-cephalad significantly, and it is an easy risk-mitigating measure concerning accidental dural puncture [8]. The skin is prepared with the neonate in a lateral decubitus position, and a sterile field is created from the anus to the shoulder. A blunt-tipped 20-gauge Crawford needle (Figure 1) is advanced through the sacrococcygeal ligament at a 45-degree angle. Once a distinct “pop” is felt, the approach angle is dropped, and the needle is advanced <1 cm. The blunt tip of the Crawford needle affords protection against dural puncture, which is a concern given the shallow depths of the thecal sac in neonates [9].

Following Crawford needle placement, the epidural catheter is used as an external “ruler” to measure the expected catheter length needed to reach the goal vertebral level. Doing so speeds up the localization of the catheter tip by ultrasound scanning. The goal vertebral level is determined using palpable anatomical approximations commonly known to correspond with various dermatomes. These anatomical approximations (Table 1) provide a reference without fluoroscopic imaging.

**Table 1:** Vertebral levels and the corresponding dermatomal distribution. Bony landmarks and corresponding vertebral levels are noted to change when placing neonates in the fetal position. Landmarking is dependent on an anatomically neutral patient, pre-positioning. Assessing levels before epidural placement is a fundamental principle for the trainee in anesthesiology.

Spinous process	Corresponding bony or dermatomal landmark
T3	Spinous process of the scapula
T4	Nipples
T7	Inferior angle of the scapula
T10	Umbilicus
L1	Inguinal
L4	Iliac crest

Next, based on the aforementioned external measurement, the styletted epidural catheter is threaded through the Crawford needle to 3-5 cm beyond the expected depth. Doing so ensures that if any segment of the epidural catheter is visualized on ultrasound at the goal vertebral level, the catheter tip is reliably cephalad. Scanning with a sterile ultrasound probe is optimal in the right and left paramedian (i.e., translaminar) probe orientations, with the long axis of the ultrasound probe parallel to the spinal column. Upon visualization, the Crawford needle is removed, and the catheter is withdrawn until the tip passes into the field of view.

**Figure 2:**
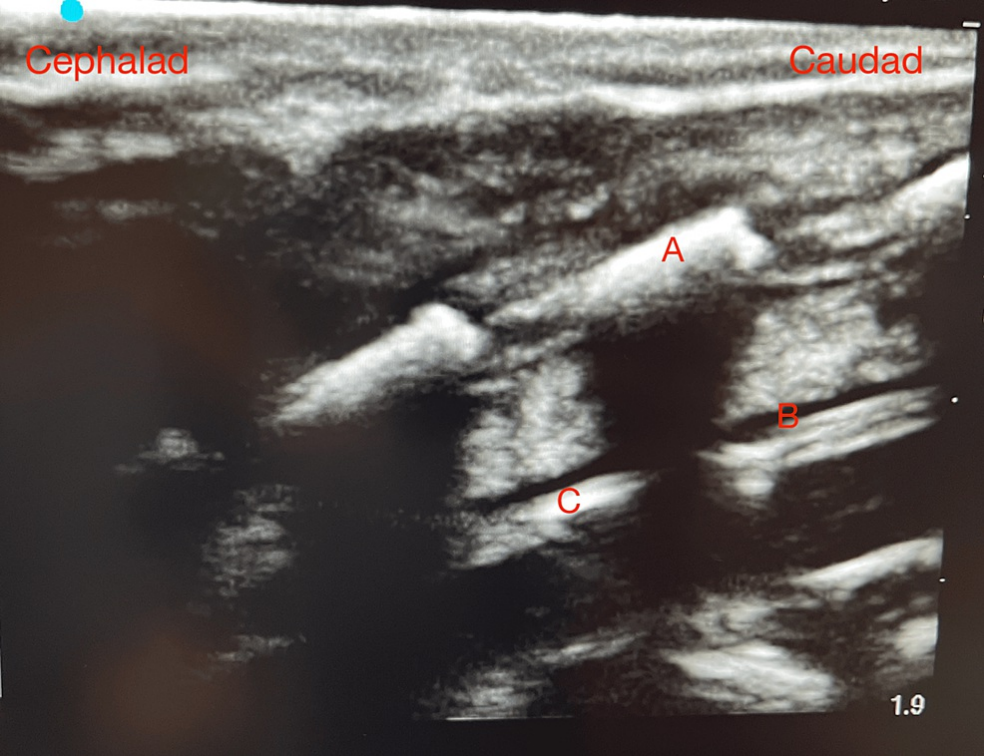
Ultrasound visualization of epidural needle placement. The different anatomic locations seen during ultrasound guidance insertion of the epidural catheter. The following landmarks were identified: (A) thoracic lamina, (B) epidural fluid, and (C) tip of the epidural catheter.

Though epidural tip localization using ultrasound is generally simple in the neonatal spine due to widely spaced vertebrae, secondary confirmation can be obtained by removing the stylette and injecting preservative-free normal saline under ultrasound visualization. Confirmatory injection results in depression of the dura and hypoechoic expansion of the epidural space (Figure 2). A weight-based test dose of local anesthetic containing epinephrine (0.1 mL/kg of 1.5% lidocaine with epinephrine, 1:200,000) is recommended at this point, given what would be potentially devastating consequences (seizure, cardiac arrest) of inadvertent intravascular injection of longer-acting amide local anesthetics [10].

Resistance to catheter insertion generally corresponds with coiling in the region of the lower spinal segments or failure to enter the epidural space to begin with. Both problems can typically be addressed by re-positioning the Crawford needle or manipulating the catheter in the lumbosacral region under ultrasound guidance if the catheter can pass into the epidural space. If manipulation by retraction is required, the Crawford needle should be removed before doing so to prevent shearing.

**Figure 3:**
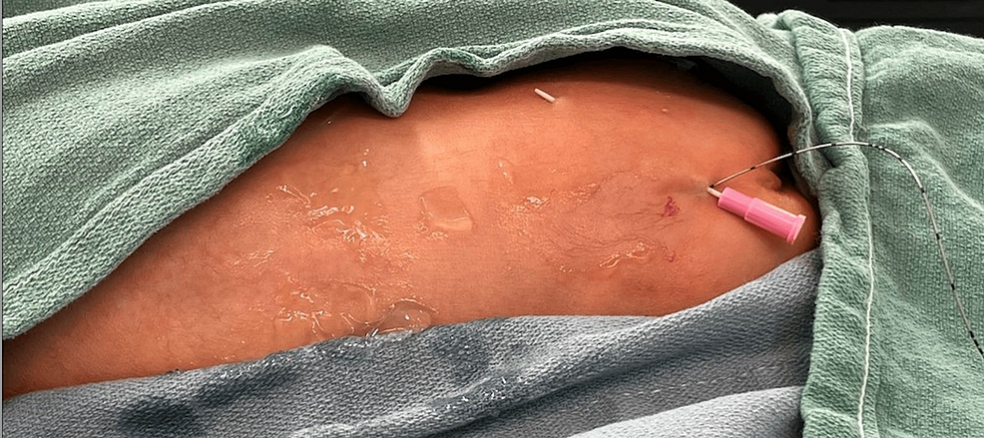
Tunneling of the epidural catheter. A subcutaneous tunnel between the skin insertion site and the epidural space where the catheter is placed. This technique reduces the risk of infection by minimizing the direct contact between the skin and the catheter.

**Figure 4:**
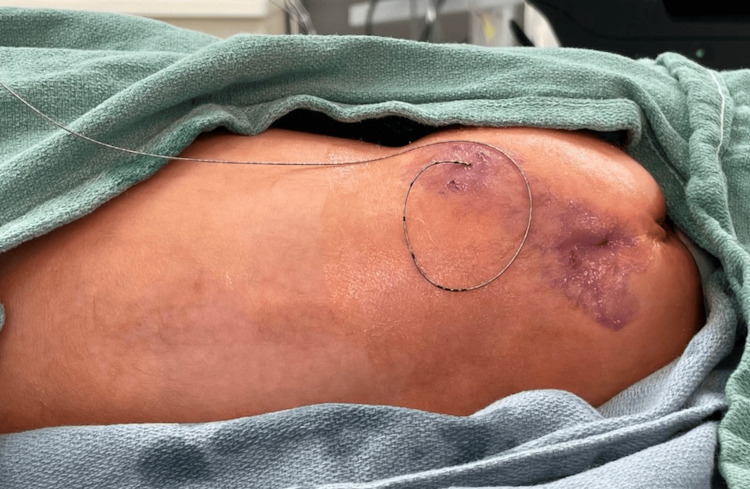
Tunnelled epidural catheter with skin glue placement.

**Figure 5:**
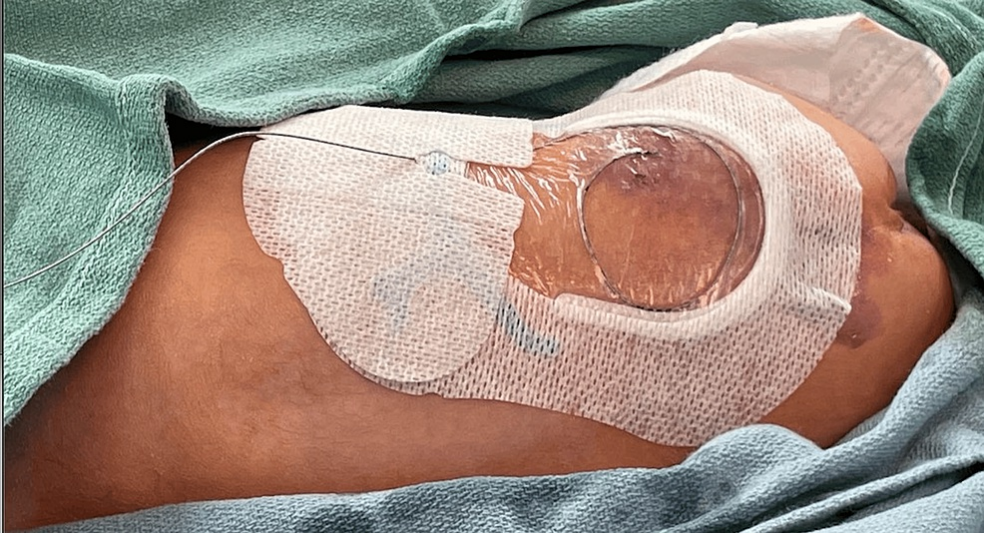
Clean and sterile dressing placement over the epidural catheter. Transparent film dressing is used to secure and protect the insertion site of the catheter. It provides a barrier against infectious organisms and contaminants while allowing visualization of the catheter insertion site.

With epidural tip location confirmed by the expected length of the catheter in the body, ultrasound visualization of epidural space expansion, and negative test dosing, attention is turned to protecting the caudal insertion site from leakage and fecal contamination. Both problems are well-addressed by applying sterile skin glue liberally to the insertion site. After sufficiently drying the glue, a foam pressure dressing is applied directly to the catheter’s insertion site. The downside of a pressure dressing is that the insertion site cannot be directly visualized for signs of infection. On the other hand, the advantage of this approach is that the combination of skin glue and pressure dressing makes insertion site complications such as contamination and leakage rare, obviating the need for frequent examination. Lastly, a reinforced transparent dressing (Sorbaview™) is applied with care to seal the inferior aspect to prevent fecal contamination. An additional transparent dressing can be helpful to seal the edges of the Sorbaview™ further if the seal is incomplete due to back curvature or skin folds. Examples of the standardized skin preparation and final dressing are shown in Figures 3-5.

Maintenance

We found data lacking in determining the ideal infusion for the maintenance of analgesia. We, therefore, determined to set standardized solutions for our neonatal critical care population that would mitigate the risk of two primary concerns, namely, motor block and local anesthetic systemic toxicity (LAST). Motor block from a high thoracic epidural following tracheoesophageal fistula repair, for example, would likely preclude extubation or result in respiratory failure after extubation. To avoid a motor block, we chose ropivacaine as the standard solution. Ropivacaine spares motor function with concentrations as high as 0.3% and has a minimally effective analgesic concentration of 0.092% in laboring women [11,12]. We, therefore, calculated a four-hour maximum dose of 0.25% bupivacaine (1 mL/kg) and converted it to a 0.1% ropivacaine equivalent on a milligram-to-milligram basis, yielding a final infusion rate of 0.5 mL/kg/hour. The 0.1% concentration was ideal due to the simplicity of dilution from standard manufactured concentrations (0.2% or 0.5%) and safe, with animal studies showing 1.5-2.5 times less risk of LAST than bupivacaine [13]. This improved safety profile was critical given the particular susceptibility of neonates to LAST. Alpha-1 acid glycoprotein is an acute-phase protein that binds amide local anesthetics and reduces the free fraction. As an acute-phase protein, neonatal levels nearly double with the stress of vaginal delivery and increase proportionately with gestational age [14]. Therefore, premature neonates and those delivered via cesarean section are at a particularly increased risk of LAST, which is why we endeavored to standardize the lowest concentration necessary. Our experience suggests that 0.1% ropivacaine is, in fact, effective for analgesia in neonates when epidural catheters are placed at a vertebral level that corresponds with the surgical level(s).

As an alternative to 0.1% ropivacaine, we standardized 1.5% 2-chloroprocaine (0.75 mL/kg/hour) to be used for patients in whom motor block is preferred (e.g., bladder exstrophy repair) or not a concern (e.g., lumbosacral catheter positions). The higher hourly volume of 2-chloroprocaine additionally provides an option for improved analgesia when the catheter tip is remote to the desired vertebral level. LAST is of little concern as 2-chloroprocaine is metabolized within seconds of entering the plasma.

With both ropivacaine and 2-chloroprocaine, we have found complementary local anesthetic solutions that meet the analgesic needs of our neonatal population with no observed morbidity related to maintenance infusions. The concern of accidental misuse of the 24-gauge epidural catheter is mitigated because syringe flushing of such a high-resistance catheter is very difficult, and the injection hub is significantly distinct from any other access hub available. Each catheter is labeled at the injection hub with a yellow caution sticker indicating the epidural location and magnetic resonance incompatibility. Lastly, all infusions are set to be non-titratable. These barriers are critical given the novelty of neonatal epidural infusions in our hospital and the safety concerns that Wong et al. identified regarding drug error risk [7].

## Discussion

Using ultrasound-guided placement of caudally inserted epidural catheters, standardized dressings, and infusions has made epidural analgesia a reliable option for our neonatal population. With almost complete spinal canal visualization possible by ultrasonography, we have found that most catheters can be directed easily and re-directed to reach the desired vertebral level. Once the catheter is confirmed to be in the epidural space on ultrasound, the Crawford needle should be removed from the patient before manipulating the catheter. Not doing so risks catheter shearing. Fortunately, the spring-wound catheter does confer some protection against complete shearing, but the concern remains. If catheter advancement is a challenge through the Crawford needle, one can consider placing an appropriately sized caudal angio-catheter (20-gauge), allowing catheter retraction without the risk of shearing. Alternatively, a lumbar insertion site using a Tuohy needle and standard loss of resistance technique will generally allow advancement to thoracic levels under ultrasound guidance. The main complication we have faced with placing epidural catheters using the caudal approach is placement failure. This failure usually arises due to the anterior axis of the sacral canal relative to the lumbar spine causing the catheter to become lodged in the anterior epidural space. However, ultrasound guidance can help correct the approach and facilitate posterior epidural space advancement. Additionally, using blunt-tip Crawford needles has prevented any occurrences of dural punctures or epidural hematomas.

**Figure 6:**
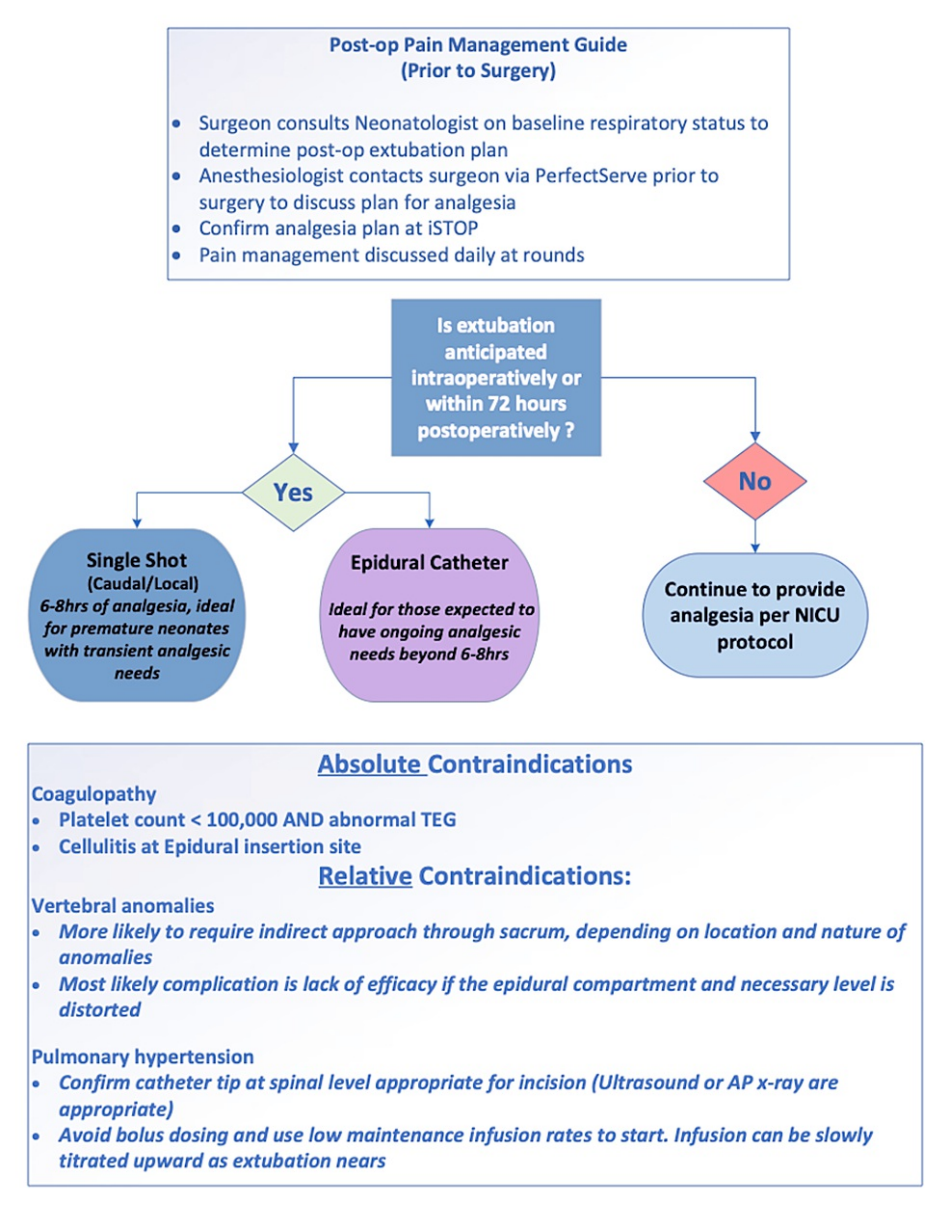
Neonatal perioperative pain management guide.

This initiative offered extensive opportunities to educate pediatric surgeons, pediatric anesthesiologists, neonatologists, and neonatal intensive care nursing staff. As detailed here, education focused on relative and absolute contraindications to epidural analgesia and measures to avoid LAST and high motor block. Bedside signage regarding the possible side effects of thoracic-level epidurals was created and included detailed information on whom to call during days, nights, and weekends. We also designed a flowsheet (Figure 6) to give all care team members a framework for conceptualizing epidural analgesia. This flowsheet underscores the primary value of reducing the need for systemic opioids and ventilator support by providing excellent analgesia with local anesthetics. Education and protocolization of neonatal epidurals have improved the consistency of implementation across our pediatric anesthesiology practice. It also has the added benefit of reducing semi-urgent communications regarding site leakage and confusion over what constitutes safe care of a neonate with an epidural.

## Conclusions

This project has allowed our team to systematically investigate epidural analgesia’s impact on this challenging patient population. Implementing our protocol has noted a decreased use of ventilator use and minimized the use of opioids. The regular use of ultrasound has significantly facilitated the objective placement of epidural anesthetics, giving clear visualization of the catheter during placement.

Having established a reliable methodology for placing neonatal epidurals, we plan to explore metrics of days of mechanical ventilation, total opioid consumption, return of bowel function, and length of stay for neonatal operations known to have difficult postoperative analgesia (e.g., thoracotomy and open upper abdominal surgeries). This data will be described in a retrospective, observational study, as there is a lack of literature regarding neonatal epidural analgesia.
